# Supplementary material for: Effect of Intermediate Airway Management on Ventilation Parameters in Simulated Pediatric Out-of-Hospital Cardiac Arrest: Protocol for a Multicenter, Randomized, Crossover Trial
Source: Children (Basel). 2023 Jan 12;10(1):148. doi: 10.3390/children10010148 (PMC9856669; doi:10.3390/children10010148)
Supplement: Supplementary file 1 [file children-10-00148-s001.zip › Supplementary File S2_Email template.pdf]

File S1. Email template.

Chères, chers collègues,

Dans le cadre de projets de recherche portant sur l'utilisation de l'i-gel / la RCP (https://swiss-cpr-studies.ch/), nous travaillons actuellement sur deux recherches conjointes (i-gel dans la réanimation pédiatrique et utilisation d'un moyen de rétrocontrôle pour optimiser la qualité du MCE). Nous avons besoins d'ambulancier-ères !

Votre service a répondu présent et nous nous réjouissons de réaliser ce projet avec vous !

En acceptant de prendre part à ce projet, vous participerez à plusieurs situations par équipes de deux générées de manière aléatoire. Le temps d'exercice total par binôme sera d'une heure et trente minutes. Vous aurez chacun·e une demi-heure d'entraînement en plus des trois ateliers destinés à l'enregistrement des données.

De plus amples informations vous seront transmises le jour de l'étude. Un temps destiné à vos éventuelles interrogations est également prévu.

Si vous souhaitez participer à la reconnaissance et l'évolution de la profession ambulancière, n'hésitez plus et participez à notre étude ! Ces exercices de réanimation adulte et pédiatrique servent également de formation continue. Vous aurez l'occasion durant cette journée de réaliser des interventions sur des mannequins haute-fidélité qui permettent une mise en situation très proche de la réalité du terrain.

En acceptant de participer, vous nous autorisez à utiliser les données enregistrées à des fins de recherche uniquement. Nous nous engageons à protéger et à anonymiser les données utilisées pour ces études. Vos données personnelles ne seront jamais transmises à des tiers, et les résultats individuels ne pourront pas être liés à votre identité.

En vous remerciant pour votre investissement, nous vous souhaitons un bon exercice de réanimation !

L'équipe de projet

Loric Stuby*, Elisa Mühlemann, Sylvain Simonet, Laurent Jampen, David Thurre, Johan N. Siebert, Laurent Suppan

*Contact investigateur principal : l.stuby@gt-ambulances.ch
